# Morphologic Findings in the Cerebral Cortex in COVID-19: Association of Microglial Changes with Clinical and Demographic Variables

**DOI:** 10.3390/biomedicines11051407

**Published:** 2023-05-09

**Authors:** Anastasiya S. Babkina, Mikhail Ya. Yadgarov, Maxim A. Lyubomudrov, Irina V. Ostrova, Alexey V. Volkov, Artem N. Kuzovlev, Andrey V. Grechko, Arkady M. Golubev

**Affiliations:** 1 Federal Research and Clinical Center of Intensive Care Medicine and Rehabilitology, Moscow 107031, Russia; mikhail.yadgarov@mail.ru (M.Y.Y.); mlyubomudrov@fnkcrr.ru (M.A.L.); iostrova@fnkcrr.ru (I.V.O.); artem_kuzovlev@fnkcrr.ru (A.N.K.); avg-2007@ya.ru (A.V.G.); arkadygolubev@mail.ru (A.M.G.); 2 Department of Pathological Anatomy, Institute of Medicine, Peoples’ Friendship University of Russia, Moscow 117198, Russia; alex.volkoff@gmail.com

**Keywords:** SARS-CoV-2, pathology, autopsy, COVID-19, brain, morphological changes, microglia, Iba-1, NeuN

## Abstract

Despite the enormous interest in COVID-19, there is no clear understanding of the mechanisms underlying the neurological symptoms in COVID-19. Microglia have been hypothesized to be a potential mediator of the neurological manifestations associated with COVID-19. In most existing studies to date, morphological changes in internal organs, including the brain, are considered in isolation from clinical data and defined as a consequence of COVID-19. We performed histological immunohistochemical (IHC) studies of brain autopsy materials of 18 patients who had died from COVID-19. We evaluated the relationship of microglial changes with the clinical and demographic characteristics of the patients. The results revealed neuronal alterations and circulatory disturbances. We found an inverse correlation between the integral density Iba-1 (microglia/macrophage-specific marker) IHC staining and the duration of the disease (R = −0.81, *p* = 0.001), which may indicate a reduced activity of microglia and do not exclude their damage in the long-term course of COVID-19. The integral density of Iba-1 IHC staining was not associated with other clinical and demographic factors. We observed a significantly higher number of microglial cells in close contact with neurons in female patients, which confirms gender differences in the course of the disease, indicating the need to study the disease from the standpoint of personalized medicine.

## 1. Introduction

Coronavirus disease 2019 (COVID-19), caused by the SARS-CoV-2 virus, was declared a pandemic by the World Health Organization (WHO) in March 2020 [1] following its outbreak in Wuhan, China. To date, more than 6.8 million fatal cases of COVID-19 have been reported [2]. Despite the enormous interest in COVID-19, many questions about the pathogenesis of the disease and its complications remain unanswered. The mechanisms underlying the neurological signs and symptoms in COVID-19 are still unclear.

According to various studies, 36–69% of COVID-19 patients suffer from anosmia, headache, dizziness, seizures, impaired consciousness, and acute cerebrovascular events, suggesting the involvement of the nervous system in the pathogenesis of COVID-19 [3,4]. In addition, neurological manifestations are common in patients after recovery from COVID-19 [5,6]. Whether neurological complications are a direct consequence of the neurotropism of the virus or due to hypoxia resulting from respiratory failure and associated coagulopathy remains unclear.

Three major routes of entry of SARS-CoV-2 into the central nervous system (CNS) have been described, including the olfactory tract [7], a hematogenous route and through the nerve endings of the cranial nerves that innervate the respiratory tract [8]. Several studies have considered the potential neurotropic effect of SARS-CoV-2 based on similar properties found in other coronaviruses [9], cases of SARS-CoV-2 detection in cerebrospinal fluid of patients with COVID-19 and autopsy materials from patients who died from COVID-19 [10,11,12], and experimental studies [13,14]. However, the detection of viral ribonucleic acid (RNA) or viral particles in the CNS does not prove their role in pathogenesis. Postmortem examination is mandatory to achieve the most complete understanding of the patterns of pathologic processes and mechanisms of CNS damage in COVID-19.

At the onset of the pandemic, there was uncertainty in many countries about the safety and appropriateness of postmortem examinations of COVID-19 patients. In particular, some institutions were reported to have refused to dissect the skull for fear of spreading aerosols of viral particles by cutting the bones of the skull. All this has significantly delayed the study of the pathogenesis of COVID-19 and its complications [15,16,17,18,19,20].

Currently, many studies are available on the morphologic changes occurring in the brain with COVID-19. A keyword search including COVID-19 and brain autopsy, histology, histopathology, neuropathology returns more than 1500 articles in the PubMed database. Histologic changes include neuronophagia, an increase in the number of microglia and astroglia, satellitosis, extensive edema, focal hemorrhage, mononuclear cell infiltration, and in some cases, foci of necrosis [21,22].

Activation of microglia is one of the most frequently detected signs of COVID-19.

Thakur K.T. et al., in a series of 41 postmortem cases, found activation of microglia in 80.5% (34/41), which confirms its important role in the pathogenesis of brain damage in COVID-19 [23,24]. SARS-CoV-2 has been reported to directly infect human microglia, resulting in cytopathic effects and microglial apoptosis [25]. An increase in the expression of Ionized calcium binding adaptor molecule 1 (Iba-1), a marker of microglia, has been revealed [26]. In addition to microglial activation, some studies have reported a tendency to increase neuronophagia [27,28]. Microglia have been hypothesized to be a potential mediator of the neurological manifestations associated with COVID-19 [25]. In particular, the leading role of microglia and mitochondrial dysfunction in the pathogenesis of long-term psychiatric and neurological disorders after COVID-19 has been suggested [29].

In most studies published to date, morphological changes in internal organs, including the brain, have been considered in isolation from clinical data and defined as being a consequence of COVID-19. Considering that each disease has its own morphological substrate, it is necessary to pay more attention to comorbidities, complications of the underlying disease, and causes of death in patients. Since the pathological processes in COVID-19, as in any other disease, are dynamic, it is reasonable to consider the duration of the disease as a potential factor influencing morphological changes.

Objective: to analyze morphological changes in the brains of patients who have died of COVID-19 and to evaluate the possible relationship of microglial changes with clinical and demographic characteristics of the patients.

## 2. Materials and Methods

We analyzed the autopsy protocols of patients (n = 152) who died in July 2021 and February 2022 with lifetime confirmation of infection with the new 2019 coronavirus (COVID-19) as the primary cause of death. Fatal COVID-19 cases (n = 18) meeting the following criteria were selected for immunohistochemical study: (1) no history of acute cerebrovascular accidents; (2) no neurodegenerative disease; (3) no chronic cerebral ischemia; (4) time interval from death to autopsy not more than 24 h.

Autopsies were performed at the Department of Pathology of the E.O. Mukhin State Clinical Hospital of the Moscow Public Health Service. Brainsamples from the parietal cortex for histological examination were taken during the pathological examination in accordance with the current legislation of the Russian Federation to verify the pathological diagnosis and to clarify the cause of death. The study protocol was approved by the local ethics committee (No. 2/21/3). According to the current guidelines of the Ministry of Health of the Russian Federation, organ samples were fixed in neutral 10% formalin solution for at least 24 h [30,31]. Formalin-fixed, paraffin-embedded tissues underwent standard processing to provide sections stained with hematoxylin and eosin, Luxol fast blue (Luxol Fast Blue Stain Kit, Abcam, Cambridge, UK). The histologic preparations were examined with a Nikon Eclipse Ni-U microscope (Nikon Instruments Inc., Tokyo, Japan).

For immunohistochemical (IHC) study, sections were deparaffinized in xylene and successively dehydrated in alcohol. High-temperature antigen retrieval was performed in citrate buffer pH 6 (Target Retrieval Solution, DAKO, Glostrup, Denmark). Sections were cooled, washed in three changes in distilled water and incubated in 3% hydrogen peroxide solution to suppress endogenous peroxidase activity. After that they were placed under Coverplates (Shandon Coverplate, Thermoscientific, Runcorn, UK) and washed with phosphate buffer (PBS IHC Wash Buffer + Tween, Cell Marque, Rocklin, CA, USA) (3 × 5 min). A blocking serum (Protein Block Serum-free, Abcam, Cambridge, UK) was used for 15 min to prevent non-specific binding of primary or secondary antibodies to tissue proteins. The sections were then incubated for 1 h at 37 °C with recombinant antibodies against the neuronal protein NeuN (1:200 dilution in Antibody Diluent, Abcam, Cambridge, UK) and rabbit polyclonal antibodies against the microglial marker protein Iba-1 (1:200 dilution in Antibody Diluent, Abcam, Cambridge, UK). Sections were washed with PBS (2 × 5 min). The reaction was visualized using the mouse- and rabbit-specific HRP/DAB (ABC) detection IHC kit (Abcam, Cambridge, UK). After washing in PBS, the sections were stained with hematoxylin. After washing in tap water, the sections were coverslipped with ImmuMount water-soluble medium (Thermo Shandon, Pittsburgh, PA, USA).

The number of microglia and neurons stained for Iba-1 and NeuN, respectively, the number of microglial cells in close contact with neurons, and the area of microglial soma were estimated on digital images (10 images from each preparation, ×200) taken using a Nikon Eclipse Ni-U microscope, digital camera, and NIS-Elements BR (Basic Research) Version 5.20 software (Nikon, Tokyo, Japan). The integral optical density (product of area and mean gray value) of microglia staining on Iba-1 was calculated using ImageJ image analysis software. The mean value per preparation was then calculated for each parameter.

The factors that could affect the morphological changes in microglia stained for Iba-1 and the number of neurons stained for NeuN (NeuN+) were sex, age, disease duration, pandemic period (year of death), presence of competing disease, presence of cerebral basilar artery stenosis, severity of lung involvement (semiquantitative assessment of CT data), lung ventilation, comorbidity (diabetes, chronic obstructive pulmonary disease, obesity), bacterial pneumonia.

The Shapiro–Wilk test was used to assess normality of the data. Continuous variables were presented as medians and interquartile ranges (IQRs), and categorical variables were described as frequencies and percentages. Group differences were assessed using the Mann–Whitney U test for continuous variables and the chi-squared test and Fisher’s exact test for categorical baseline variables. The Spearman rank correlation coefficient was used as a hypothesis test to examine the relationship between continuous variables. All analyses were performed using IBM SPSS Statistics for Windows, Version 27.0. Armonk, NY, USA: IBM Corp. The significance level was set at 0.05.

## 3. Results

Fatal complications among those who died from COVID-19 in July 2021 (n = 99) included respiratory failure in 80 cases (81%), cardiopulmonary failure in five cases (5%), and pulmonary embolism in 14 cases (14%). Chronic cerebral ischemia was found in three cases (3%), hemorrhagic stroke in one case (1%), and autopsy was performed more than 24 h after death in 84 cases. Thus, 11 patients out of 99 met the inclusion criteria for the IHC study.

Fatal complications among those who died from COVID-19 in February 2022 (n = 53) included respiratory failure in 37 patients (70%), cardiopulmonary failure in nine patients (17%), acute heart failure in four patients (7%), and cerebral edema in three patients (7%). Ischemic stroke was noted in four cases (7%), chronic cerebral ischemia in two cases (4%), and senile degeneration of the brain in three cases (6%). Of the remaining 44 cases, autopsies were performed more than 24 h after death in 37 cases. Thus, out of 53 patients, seven patients met the inclusion criteria for the IHC study.

In total, out of 152 deaths analyzed, acute cerebrovascular accidents (ACVA) were recorded in five patients, with ischemic stroke diagnosed in four patients (3%) and hemorrhagic stroke in one patient. A summary of patients with ACVA is shown in Table 1.

Data on the COVID-19 non-survivors included in the studies using the IHC method are shown in Table 2 and the Supplementary Materials. Of the 18 cases, the immediate causes of death included respiratory failure in 15 patients, cardiopulmonary failure in two, and heart failure in one patient.

**Figure 1:**
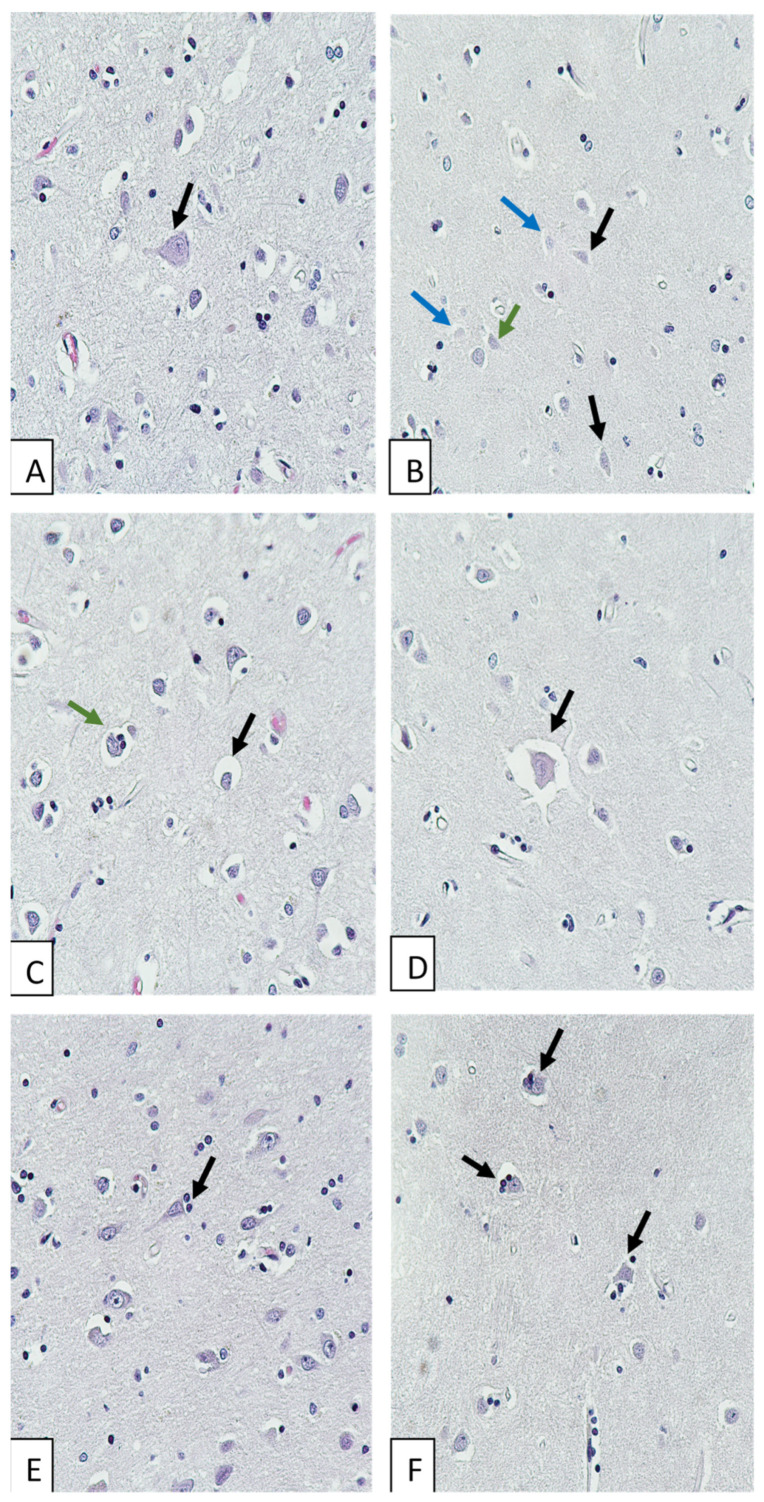
Morphological changes in the cerebral cortex of COVID-19 non-survivors. Hematoxylin and eosin staining. Magnification, ×400. (**A**) Neuronal swelling. (**B**) Ghost cells (shown by blue arrows), hypochromic neurons (shown by black arrows), and karyolysis (green arrow). (**C**) Pericellular edema (shown by black arrow); neuronophagia (shown by green arrow). (**D**) Severe pericellular edema. (**E**) Sattelitosis. (**F**) Neuronophagia.

Morphological study of brains of COVID-19 non-survivors revealed neuronal damage: acute swelling (Figure 1A), eccentricity of nuclei and nucleoli, karyocytolysis, ghost cells (Figure 1B), neuronophagia and sattelitosis (Figure 1E,F). We also observed signs of circulatory disturbances: perivascular and pericellular edema (Figure 1C,D), diapedetic hemorrhages, congestion, microvascular engorgement. Corpora amylacea were found in some brain preparations, and lipofuscin was found in the cytoplasm of some neurons.

**Figure 2:**
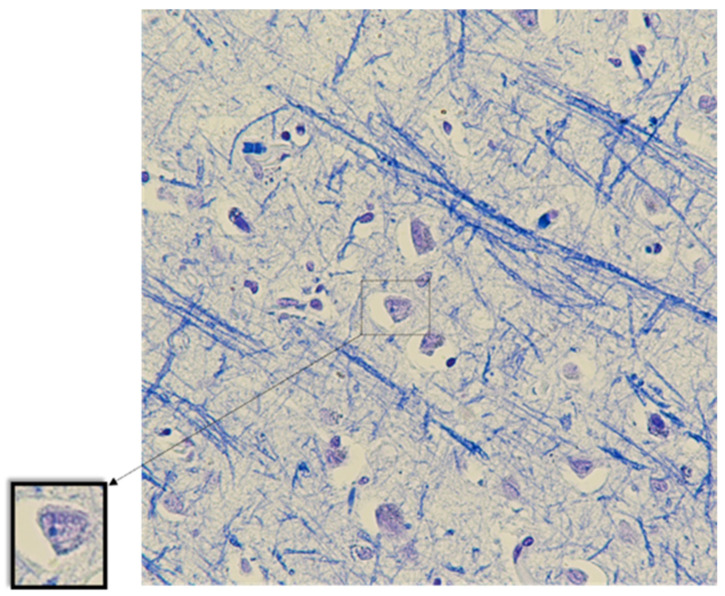
The cerebral cortex. Blue staining of neuronal nucleoli, eccentricity of nucleoli. Luxol fast blue staining. Magnification, ×400.

An irregular staining of nerve fibers was observed when stained with Luxol fast blue. Blue staining of nerve cell nucleoli was observed (Figure 2).

**Figure 3:**
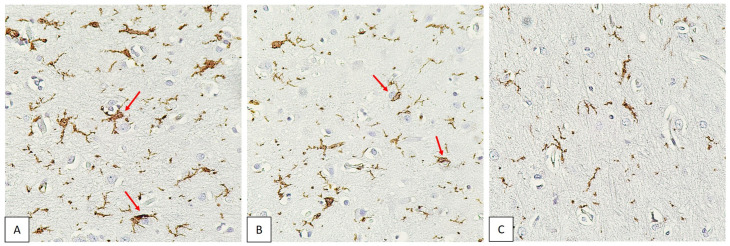
Cerebral cortex of COVID-19 non-survivors. IHC staining for the microglial marker Iba-1. Magnification, ×400. Different intensity of staining from intense to weak (**A**–**C**). Microglial cells in contact with neurons are marked with arrows.

When stained for Iba-1, microglial cells were visualized in all specimens. Visible differences in the intensity of microglial staining in the specimens were noted. Some microglial cells were in close contact with neurons (Figure 3).

When stained for NeuN, no positively IHC-stained cells were found in three specimens. These specimens were stained repeatedly. However, the staining results remained the same.

**Figure 4:**
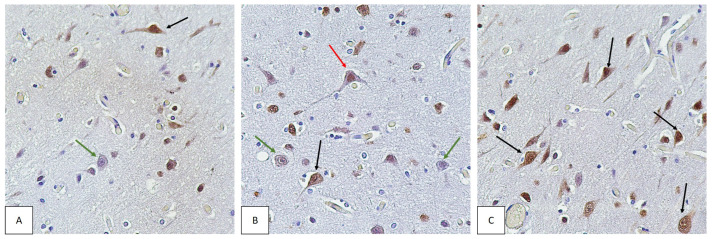
Cerebral cortex of COVID-19 non-survivors. IHC staining for the neuronal marker NeuN. Magnification, ×400. Different localization of NeuN in the neuron. NeuN localized in the neuronal nucleus is indicated by red arrows (**B**). NeuN localized in the nucleus and cytoplasm of neurons is indicated by black arrows (**A**–**C**). Neurons not stained for NeuN are indicated by green arrows (**A**,**B**).

Some neurons showed no IHC staining. In the vast majority, positive staining was observed not only in the nucleus, but also in the cytoplasm of neurons (Figure 4).

When analyzing the relationship between morphological changes in microglia, NeuN+ neurons in the cerebral cortex of patients who died of COVID-19 and the clinical and demographic characteristics of the patients, we obtained the results shown in Table 3.

A significantly higher number of female patients (≥3, median) with microglia in direct contact with neurons was found (*p* = 0.02).

**Figure 5:**
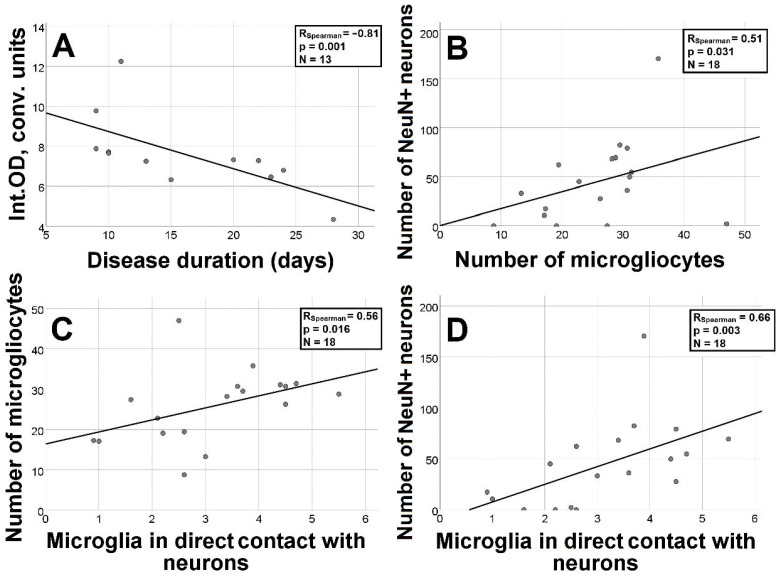
Graphs characterizing the relationship between the integral density of microgliocytes stained for Iba-1 and disease duration (**A**); the number of microgliocytes and the number of NeuN+ neurons (**B**) and the number of microgliocytes in contact with neurons (**C**); and the number of NeuN+ neurons with the number of microglial cells in contact with neurons (**D**).

Integral density of Iba-1 staining was significantly lower in patients with disease duration longer than 14 days (*p* = 0.03) (Table 3). The correlation between disease duration and integral density of Iba-1 staining was strongly negative (R = −0.81, *p* = 0.001) (Figure 5A).

The number of NeuN+ neurons and microglial characteristics such as their number and soma area were not associated with any of the clinical and demographic factors.

When the correlations between the number of NeuN+ neurons and microglial characteristics were analyzed, the number of NeuN+ neurons was positively correlated with the number of microglial cells (R = 0.51, *p* = 0.031) (Figure 5B), including those in close contact with neurons (R = 0.66, *p* = 0.003) (Figure 5D). The number of microglial cells positively correlated with those in close contact with neurons (R = 0.56, *p* = 0.016) (Figure 5C). Microglial soma area did not correlate with other characteristics.

## 4. Discussion

Our study revealed morphological changes in the brain during COVID-19, such as neuronal alterations and circulatory disturbances. When analyzing the relationship between microglial changes and clinical and demographic characteristics of the patients, we found an inverse correlation between the integral density of Iba-1 staining and the duration of the disease. The integral density decreased with disease duration over 14 days. We showed a significantly higher number of microglial cells in close contact with neurons in female patients. The results obtained by analyzing the relationship between microglia and neurons stained for NeuN show a positive correlation between the number of neurons stained for NeuN and the number of microglial cells, including those in close contact with neurons.

### 4.1. Ischemic Stroke in COVID-19

For the selection of material for the IHC study, we analyzed 152 pathology protocols. Ischemic stroke was detected in four patients (3%). Based on our results, we cannot claim that ischemic stroke is a common complication of COVID-19. According to Luo et al., the cumulative prevalence of ischemic stroke in COVID-19 was 2% (95% CI: 1–2%; *p* < 0.01) [32], which is similar to our results. Given the patients’ severe comorbidity and risk factors such as hypertension, atherosclerosis, basal cerebral artery stenosis, and a history of ischemic stroke in two of four patients, it is difficult to determine whether stroke is a complication of COVID-19. Respiratory failure in COVID-19 certainly worsens the initial status of the patient but may be associated with other pulmonary diseases. The study by Beach et al. aimed at comparing the incidence of ischemic and hemorrhagic stroke in patients with pneumonia and COVID-19 with that of ACVA in other pneumonias not associated with COVID-19 showed a similar risk of ACVA in patients of both groups [33]. The role of associated coagulopathies cannot be excluded. Out of five patients with ACVA, thrombotic complications were found in three patients.

### 4.2. Evidence of Brain Damage in COVID-19

The morphological changes in the brain described in the present study are probably caused by hypoxic brain damage due to respiratory failure, circulatory disorders, or coagulopathies associated with COVID-19. Similarly, P. Canoll et al. analyzed 41 cases of COVID-19 deaths and found neuronal damage in the brain caused by hypoxia in all cases [23].

The large number of neurons with eccentric nuclei and nucleoli was remarkable in our study. This feature was most often associated with acute neuronal swelling. According to McIlwain et al., nuclear eccentricity is associated with structural changes in the cytoskeleton caused by axonal damage [34]. Damage to axons may be due to their compression by edema (pericellular edema of varying severity was found in all cases examined). Luxol fast blue staining of neuronal nucleoli was described by Dahmen and Müller in cases of intracranial hypertension. The authors attributed this phenomenon to a lactacidotic pH shift caused by cerebral hypoxia [35]. The observed signs of karyocytolysis and neuronophagia may be caused by both hypoxic injury to neurons and possible neuronotropic and deleterious effects of SARS-CoV-2 virus, which cannot be ruled out based on our results.

### 4.3. Neurotropic Effect of SARS-CoV-2 Virus

The ability of SARS-CoV-2 virus to infect and damage brain cells has been described in several experimental studies. Villadiego et al. observed high-intensity SARS-CoV-2 nucleocapsid staining, predominantly in neurons of the cortex, olfactory bulbs, basal forebrain, amygdala, thalamus, hypothalamus, and mesencephalon in susceptible transgenic mice 6 days after infection. Stereological quantification of hypothalamic and cortical neurons showed a significant decrease in neuronal density in SARS-CoV-2-infected mice on days 4 and 6 after infection [36]. Beckman et al. detected SARS-CoV-2 in olfactory brain neurons of macaques 7 days after infection. This was followed by activation of astroglia and microglia and direct contact between infected neurons and glial cells, suggesting that infection induces a response that may contribute to neuronal death [37].

Most of the studies confirming the direct damaging effects of the virus are experimental. Caution should be exercised in extrapolating experimental data to humans. Studies that have detected viral RNA or particles in the brains of COVID-19 deceased patients do not support a role for SARS-CoV-2 in the pathogenesis of neurological complications. Cama et al. used confocal immunofluorescence spectroscopy to detect viral nucleocapsid protein in neurons, astrocytes, oligodendrocytes, and microglia of patients who died of COVID-19 [24]. Song et al. showed the presence of viral spike protein in cortical neurons and in the microcirculation in the brains of three patients with COVID-19 who died [10]. Thakur et al. observed insignificant titers of viral RNA in brain samples from 28 of 41 patients who died of COVID-19 [23]. In a study by Matschke J. et al., SARS-CoV-2 RNA or proteins (spike or NC) were detected in 21/40 (53%) patients, with both viral components present in 8/40 (20%) cases, but their presence in the CNS was not associated with the severity of morphologic changes [11]. The study by Stein et al. showed the spread of SARS-CoV-2 RNA to all organs, including the brain. However, histologic changes characteristic of inflammation or evidence of direct cytopathic effect of the virus outside the respiratory tract were not seen [38].

Eschbacher K.L. et al. examined autopsy brain material from 50 people who died of COVID-19 and found morphological changes typical of the age group of the patients and did not detect SARS-CoV-2 RNA in any of the samples [39].

Therefore, the data obtained from autopsy materials are inconsistent, and cannot clearly confirm or refute the hypothesis of neurotropic effects of the virus.

### 4.4. Functions and Diagnostic Significance of the NeuN Protein

In the present study, immunohistochemical staining for the neuronal nuclear protein NeuN was used to visualize neurons and assess microglia–neuron relationships. This neuron-specific nuclear protein stably expressed in most postmitotic neurons of the vertebrate nervous system. The absence of NeuN staining has been considered as a marker of neuronal damage in several studies [40,41,42,43].

Studies using primary antibodies against NeuN have been performed on both experimental and human autopsy material. Luijerink L. et al. found that the IHC reaction was weaker when staining human brain sections fixed in formalin and embedded in paraffin than when staining brains from experimental animals. The authors attributed the reduction in staining to the influence of fixation and histological preparation technique [44]. However, studies on experimental material show a high variability in neuronal staining independent of material fixation [45].

Many studies have questioned the role of NeuN as a marker of uninjured neurons, pointing out that staining is variable and may be absent in certain diseases and physiological conditions [46]. Later, NeuN was found to be an epitope of Rbfox3, a member of the Rbfox1 splicing factor family [47], and the presence or absence of IHC reaction with primary antibodies against NeuN was shown to depend on the phosphorylation status of this epitope. Consequently, the absence of NeuN staining may not be associated with neuronal death, but with a decrease in NeuN protein expression or loss of NeuN antigenicity [48]. However, the factors leading to a decrease in protein expression or loss of antigenicity are not well understood.

In our study, absence of IHC staining was observed in three cases. In the remaining cases, there were areas of both strong neuronal staining and no staining within the same specimen. Since the analysis of the number of neurons stained for NeuN did not reveal the influence of any factor, we have no reason to suggest what might cause the absence of staining in neurons.

### 4.5. The Importance of NeuN Localization in Neurons

While examining the brains of COVID-19 deceased, we noticed that NeuN was localized not only in the nucleus but also in the cytoplasm. The phenomenon of the cytoplasm staining to a lesser extent than the nucleus has previously been described as being unrelated to abnormal processes [49]. In some neurons, such as granular cells of the cerebellum, there is no nuclear staining in afferent vegetative neurons while the cytoplasm shows a positive IHC reaction [50]. The differences in IHC staining are due to the subcellular localization of different NeuN/Rbfox3 subtypes. The 46 kDa subtype is mainly distributed in the nucleus, whereas the 48 kDa subtype is mainly located in the cytoplasm [51].

NeuN staining in both the nucleus and cell bodies, including processes, was detected in the brain of a human immunodeficiency virus type I-associated neurocognitive disorder. In this disease, neuronal dysfunction is observed as a consequence of HIV-1 infection of brain macrophages and microglia [46].

Analyzing the causes of NeuN-positive staining of the cytoplasm of brain neurons of patients who died of COVID-19, we cannot exclude the association of this phenomenon with hypoxic damage to neurons and neuronal dysfunction, as in HIV-1 disease, but postmortem changes involving autolysis and abnormal permeability of biological membranes, including the nuclear one, should also be considered.

### 4.6. Functions of Microglia

Several studies have demonstrated microglial activation in COVID-19. Microglia are the primary immune cells in the central nervous system [52]. Unactivated, ramified microglia continuously scan the environment for “danger signals” associated with pathogens or injury [53,54]. Upon detection of a “danger signal”, microgliocytes undergo a rapid change in morphology and function, becoming activated and capable of phagocytosis [55]. An important function of microglia is to participate in synapse formation and pruning, as well as the regulation of neurogenesis. Thus, microglia help maintain homeostasis in the CNS and respond to changes in homeostasis by releasing a cascade of biologically active factors [56]. Microglial activation has been shown to be critical in attenuating neuronal apoptosis, enhancing neurogenesis, and promoting functional recovery after cerebral ischemia [57].

Considering NeuN as a marker of intact neurons, the positive correlation between the number of microglial cells, including those in close contact with neurons, and the number of stained neurons may indicate the protective effect of microglia.

Although microglial activation is a protective mechanism, chronic or excessive activation is implicated in the pathogenesis of many neurodegenerative and psychiatric disorders [58].

### 4.7. Role of Microglia in Viral Infections

Several factors associated with infection can affect microglial function. One such factor is the ability of some viruses (HIV, JEV, ZIKV, and SARS-CoV-2) to invade microglia and inhibit repair and regeneration processes. The second damaging factor is the excessive production of neurotoxic factors, including reactive oxygen species (ROS). ROS production by microglia has been reported in the context of brain infection with various viruses. ROS have been shown to contribute to neuronal cell death. A long-term effect of these factors on microglial function has been suggested [59].

Ultrastructural analysis has also shown that microglia exhibit increased phagocytic activity and extracellular digestion of degraded elements during infection. In a study of mice in which microglial activity was pharmacologically suppressed by PLX5622, increased Zika virus replication was observed [60].

A steady increase in extracellular ATP released from damaged neurons has been shown during viral infection, leading to activation and recruitment of microglia for phagocytosis within minutes or hours. Extracellular ATP is a potent chemotactic signal for microglia. ATP is an agonist of the microglial P2Y receptor and through its stimulation mediates the rapid response of microglia to injury [61].

Our study showed a correlation between the number of microglia and the number of direct microglial contacts with neurons, indicating microglial activation.

In a study of K3-hACE18 transgenic mice infected with SARS-CoV-2, acute production of microglial IL-2 and TNF-α was followed by chronic loss of microglia. The authors hypothesize that microglia mediate the neurological complications caused by SARS-CoV-2 [25].

We were unable to identify factors that influence microglial characteristics such as number and size. However, due to the small sample size, the influence of clinical and demographic factors on the microglial characteristics studied cannot be definitively excluded.

### 4.8. Role of Iba-1 in Microglial Activation

To visualize microglia, we used primary antibodies against ionized calcium-binding adaptor protein-1 (Iba-1). Iba-1 is an actin-cross-linking protein whose expression is observed in macrophages [62], including all types of microglia. It is widely used as an immunohistochemical marker for both ramified and activated microglia. Since microglial activation is associated with increased Iba-1 expression, as shown in several studies, Iba-1 staining densitometry can be used to measure microglial activation [58]. However, some studies argue that microglial activation in brain tissue is not always associated with increased Iba-1 expression [63].

To assess the adequacy of Iba-1 assessment, it is necessary to know the function of this protein. Microglial motility is provided by the organization of the actin cytoskeleton, which is connected to the sensory system of membrane receptors, allowing microglia to perceive changes in the microenvironment and regulate responses. Iba-1 is a protein required for actin binding and microglial membrane ruffling [64].

Results from a study of microglial activity, synaptic function, and behavior in Iba-1-deficient mice (Aif1) suggest that Iba-1 plays a critical role in microglial activation. The brains of Aif1-deficient mice were shown to have altered expression of proteins related to synaptic pruning, as well as a deficit in the number of excitatory synapses that persisted into adulthood and correlated with significant behavioral changes [65].

An experimental study in mice infected with influenza virus confirmed a significant role for Iba-1 in the regulation of microglial immunological functions during viral infection [66].

### 4.9. Changes in Iba-1 Expression in Different Diseases

Given the important function of Iba-1 in the CNS and the potential involvement of microglia in the pathogenesis of COVID-19, the decrease in integral density of Iba-1 staining as a function of disease duration demonstrated in our study deserves special attention.

The dynamic of Iba-1 expression has been followed in an experimental influenza model and was characterized by increased expression in microglial cells on day 3 after infection, peaking on day 7 and gradually decreasing to baseline levels by day 35 [66]. The peak of Iba-1 expression in the penumbra zone after transient middle cerebral artery occlusion was demonstrated 7 days after ischemia [67].

The study by Serrano G.E. et al. showed that the area occupied by Iba-1-positive microglia was significantly reduced in the amygdala and cerebellum of COVID-19 deceased patients, while no changes were detected in LN3-labeled (“activated”) microglia. The authors explain this phenomenon of COVID-19-associated immunosuppression of brain immune effector cells by drawing analogies to changes in microglia homologues in COVID-19, which include reduced numbers, gene expression and morphological abnormalities of monocytes, disrupted germinal centers and dendritic cell networks of lymph nodes [68].

Decreased Iba-1 integral optical density in COVID-19 may indicate decreased Iba-1 expression or microglial damage, depending on the duration of the disease. As the protein has an important functional role, we can assume that microglial function is impaired in long-term disease, which may lead to delayed neuropsychiatric disorders in survivors. Notably, the integral optical density index was not associated with any of the clinical and demographic factors other than disease duration. However, we cannot claim that this phenomenon is related to SARS-CoV-2 neurotropism, because COVID-19 is associated with respiratory failure. Therefore, the observed changes may be related to the duration of hypoxia and the worsening of hypoxic brain damage as the disease progresses.

### 4.10. Influence of Sex on the Pathogenesis of COVID-19

Interesting results were obtained in the analysis of factors influencing the number of microglia in close contact with neurons, i.e., active microglia. The number of microglia in close contact with neurons was found to be higher in the female group of patients.

Gender has been shown to influence the course of COVID-19, its complications and late effects [69]. Sex differences have been shown in the correlations between patient-specific clinical parameters and their global metabolic profiles [70].

Several studies have shown that female sex is a risk factor for long-term neuropsychiatric symptoms after COVID-19 [29,71,72,73].

Considering the research data on the role of microglial hyperactivation in the pathogenesis of long-term neurological disorders, as well as the results of our study, we can assume that the high frequency of delayed neurological symptoms in female patients is associated with greater microglial activity during COVID-19.

### 4.11. Study Limitations

Our study has several limitations, mainly due to its retrospective nature: (1) small sample size (n = 18) due to a small number of autopsies performed within 24 h post mortem; (2) lack of age-matched healthy controls, since this study was part of a project investigating the molecular aspects of COVID-19 pathogenesis and the researchers did not have access to autopsy material; (3) lack of data on disease duration in five cases (from disease onset) and on CT lung involvement in two cases; (4) use of only one neuronal and one microglial marker for IHC staining. A study of microglia–neuron interactions using additional IHC markers would significantly broaden the understanding of changes in the cerebral cortex of patients who died of COVID-19 and the mechanisms of brain damage. Therefore, this direction deserves further investigation.

However, these limitations did not prevent us from obtaining results demonstrating the influence of disease duration and patient sex on the morphological characteristics of microglia. The data obtained are scientifically novel and provide a rationale for studying morphological changes in internal organs in combination with clinical and demographic characteristics of patients.

## 5. Conclusions

Morphological changes in the brain during COVID-19 include neuronal alterations and circulatory disturbances. The inverse relationship between the integral density of Iba-1 staining and the duration of the disease, its decrease with the duration of the disease over 14 days may indicate a reduced activity of microglia and do not exclude their damage in the long-term course of COVID-19. Since the integral density of Iba-1 was not influenced by other clinical and demographic factors, we can assume that this phenomenon is due to the specific features of COVID-19 pathogenesis. The increased number of microglia in close contact with neurons in female patients confirms gender differences in the course of the disease, indicating the need to study the disease from the standpoint of personalized medicine.

## Figures and Tables

**Table 1:** Characteristics of patients with COVID-19 excluded from the study due to the presence of ACVA.

No	Sex	Age, y	Direct Cause of Death	Comorbidity	Type of ACVA
1	M	35	Massive thromboembolism of the right and left pulmonary arteries. Right atrial auricular thrombosis.	Hypertension with predominant cardiac involvement: heart weight 560 g, concentric myocardial hypertrophy, left ventricular wall thickness 1.8 cm. Atherosclerosis of aorta and iliac arteries (stage II, grade 1), carotid arteries (stage I, grade 1), coronary arteries (stage II, grade 2, 40% stenosis).	Intracerebral hemorrhages of hematoma type in the region of subcortical nuclei of the left cerebral hemisphere.
2	F	92	Cerebral edema with compression of the cerebellum into the foramen magnum. Severe pulmonary edema.	Hypertension with predominant heart and kidney damage. Atherosclerosis of the cerebral basal arteries (stage II, grade 2, 40% stenosis). CHD.	Ischemic infarction in all lobes of the right cerebral hemisphere with hemorrhagic component.
3	F	81	Cerebral edema. Severe pulmonary edema. Ischemic infarction of kidney (according to histologic examination)	Hypertension with predominant heart and kidney damage. Atherosclerosis of the cerebral basal arteries (stage II, grade 2, 40% stenosis), mass lesion of the dura mater of the anterior cranial fossa (angiomatous meningioma microscopically).	Ischemic infarction of the frontal lobe of the right cerebral hemisphere.
4	M	70	Cerebral edema. Severe pulmonary edema	Previous myocardial infarction of the posterior wall of the left ventricle. 80% stenotic atherosclerosis of the coronary arteries (stage IV). Hypertension with predominant heart and kidney damage. Previous acute cerebrovascular accident in the right and left cerebral hemispheres: large brown cysts in the temporal lobe of the right hemisphere, lacunar cysts in the subcortical nuclei of the right and left hemispheres, in the white matter of the temporal lobe of the left hemisphere. Atherosclerosis of the cerebral basal arteries (stage II, grade 3, 70% stenosis).	Ischemic infarction of the occipital lobe of the right cerebral hemisphere with a hemorrhagic component.
5	F	83	Pulmonary edema. Pulmonary artery thrombosis	Hypertension: Atherosclerosis of coronary arteries (fibrous plaques, stenosis up to 50%); cerebral basal arteries (fibrous plaques, stenosis up to 50%).	Secondary focus of necrosis of the left occipital lobe.

**Table 2:** Characteristics of the patient cohort in the study.

Parameters		Value
N		18
Confirmed COVID-19 diagnosis		18 (100%)
Sex (M)		9 (50%)
Age (years)		64.5 (44.8–73.3), range: 18–90
Disease duration (days), N = 13		14 (10–23), range: 9–28
Disease duration ≥ 14 days		7/13 (54%)
Concomitant/competing cause of death		5 (28%)
Year of death	2021 2022	11 (61%) 7 (39%)
Intracranial artery stenosis	0% 20% 40% 50%	7 (39%) 2 (11%) 7 (39%) 2 (11%)
Computed tomography data	CT, 1–25% involvement	3 (17%)
	CT, 26–49% involvement	3 (17%)
	CT, 50–75% involvement	3 (17%)
	CT, 76–100% involvement	8 (44%)
	No data	1 (6%)
Mechanical ventilation		9 (50%)
Hospital length of stay (days)		8 (5–10), range: 1–18
Comorbidity		
Diabetes mellitus		4 (22%)
Chronic obstructive pulmonary disease		1 (6.0%)
Coronary heart disease		8 (44%)
Hypertension		14 (78%)
Obesity		7 (39%)
Bacterial pneumonia		6 (33%)
Morphological Parameters		
Number of neurons (NeuN), N/specimen		40.7 (8.5–68.6)
Number of microgliocytes, N/specimen		27.8 (18.7–30.8)
Microglia in close contact with neurons, N/specimen		3.2 (2.2–4.4)
Integral optical density of microglia, conv. units		7.3 (6.7–8.1)
Average microgliocyte area, µm^2^		56.5 (54.4–70.6)

**Table 3:** Impact of clinical and demographic parameters, disease duration and comorbidity on morphological parameters.

Parameters	Number of Neurons (NeuN), N/Specimen			Number of Microgliocytes, N/Specimen			Microglia in Close Contact with Neurons, N/Specimen			Int. OD, conv. Units			Average Microgliocyte Area, µm^2^		
	≥40, N = 9	<40, N = 9	*p*	≥28, N = 9	<28, N = 9	*p*	≥3, N = 9	< 3, N = 9	*p*	≥7.3, N = 9	<7.3, N = 9	*p*	≥56.5, N = 9	<56.5, N = 9	*p*
Sex (M)	3, 33.3%	6, 66.7%	0.3	3, 33.3%	6, 66.7%	0.3	2, 22.2%	7, 77.8%	0.02 *	4, 44.4 %	5, 55.6%	0.9	3, 33.3%	6, 66.7%	0.3
Age (years)	62 (45–72)	71 (49–82)	0.5	62 (32–71)	71 (54–85)	0.2	62 (45–71)	71 (42–85)	0.4	70 (61–77)	47 (29–73)	0.3	70 (41–73)	62 (46–77)	0.9
Hospital length of stay (days)	9 (5–14)	6 (5–9)	0.3	6 (4–11)	8 (6–11)	0.4	7 (4–11)	8 (6–11)	0.5	6 (4–8)	10 (6–12)	0.2	6 (3–10)	8 (6–13)	0.2
Disease duration (days)	18 (11–22)	12 (10–25)	0.8	11 (10–19)	20 (10–24)	0.4	11 (9–17)	21 (12–25)	0.09	10 (9–13)	23 (15–25)	0.01 *	12 (10–25)	18 (11–22)	0.8
Disease duration ≥ 14 days	5/7, 71.4%	2/6, 33.3%	0.3	3/6, 50.0%	4/7, 57.1%	0.9	4/6, 66.7%	3/7, 42.9%	0.6	1/6, 16.7%	6/7, 85.1%	0.03 *	3/7, 42.9%	4/6, 66.7%	0.6
Concomitant/competing cause of death	4, 44.4%	1, 11.1%	0.3	4, 44.4%	1, 11.1%	0.3	4, 44.4%	1, 11.1%	0.3	3, 33.3%	2, 22.2%	0.9	2, 22.2%	3, 33.3%	0.9
Presence of intracranial artery stenosis	5, 55.6%	6, 66.7%	0.9	5, 55.6%	6, 66.7%	0.9	6, 66.7%	5, 55.6%	0.9	8, 88.9%	3, 33.3%	0.05	5, 55.6%	6, 66.7%	0.9
Year of death (2022)	3, 33.3%	4, 44.4%	0.9	3, 33.3%	4, 44.4%	0.9	4, 44.4%	3, 33.3%	0.9	3, 33.3%	4, 44.4%	0.9	3, 33.3%	4, 44.4%	0.9
CT, 76–100% involvement	4/8, 50.0%	4/9, 44.4%	0.9	5/8, 62.5%	3/9, 33.3%	0.3	5/8, 62.5%	3/9, 33.3%	0.3	5/8, 62.5%	3/9, 33.3%	0.3	5/8, 62.5%	3/9, 33.3%	0.3
Mechanical ventilation	4, 44.4%	5, 55.6%	0.9	5, 55.6%	4, 44.4%	0.9	5, 55.6%	4, 44.4%	0.9	4, 44.4%	5, 55.6%	0.9	5, 55.6%	4, 44.4%	0.9
Diabetes mellitus	3, 33.3%	1, 11.1%	0.6	3, 33.3%	1, 11.1%	0.6	3, 33.3%	1, 11.1%	0.6	2, 22.2%	2, 22.2%	0.9	2, 22.2%	2, 22.2%	0.9
Chronic obstructive pulmonary disease	1, 11.1%	0, 0%	0.9	0, 0%	1, 11.1%	0.9	0, 0%	1, 11.1%	0.9	0, 0%	1, 11.1%	0.9	0, 0%	1, 11.1%	0.9
Coronary heart disease	3, 33.3%	5, 55.6%	0.7	3, 33.3%	5, 55.6%	0.7	3, 33.3%	5, 55.6%	0.7	6, 66.7%	2, 22.2%	0.2	4, 44.4%	4, 44.4%	0.9
Hyperten-sion	7, 77.8%	7, 77.8%	0.9	6, 66.7%	8, 88.9%	0.6	7, 77.8%	7, 77.8%	0.9	8, 88.9%	6, 66.7%	0.6	6, 66.7%	8, 88.9%	0.6
Obesity	3, 33.3%	4, 44.4%	0.9	2, 22.2%	5, 55.6%	0.3	3, 33.3%	4, 44.4%	0.9	3, 33.3%	4, 44.4%	0.9	5, 55.6%	2, 22.2%	0.3
Bacterial pneumonia	2, 22.2%	4, 44.4%	0.6	2, 22.2%	4, 44.4%	0.6	3, 33.3%	3, 33.3%	0.9	3, 33.3%	3, 33.3%	0.9	1, 11.1%	5, 55.6%	0.1

* Differences are statistically significant.

## Data Availability

The data that support the findings of this study are available from the corresponding author upon reasonable request. Participant data without names and identifiers will be made available after approval from the corresponding author and local Ethics Committee.

## Supplementary Materials

The following supporting information can be downloaded at: https://www.mdpi.com/article/10.3390/biomedicines11051407/s1, Table S1: Data on the non-survivors with COVID-19 included in the studies.
